# Use of Bupropion in the Management of Negative Symptom Schizophrenia: A Case Series

**DOI:** 10.7759/cureus.23518

**Published:** 2022-03-26

**Authors:** Pradilka Perera, Ganeya Gajaram, Danish Qureshi, Manpreet Gill, Amod Thanju, Afrina Zaman, Patrice Fouron, Ayodeji Jolayemi

**Affiliations:** 1 Psychiatry, Interfaith Medical Center, Brooklyn, USA; 2 Psychiatry, Medical University of the Americas / Interfaith Medical Center, Brooklyn, USA

**Keywords:** case report series, residual, secondary negative symptoms, primary negative symptoms, panss score, adjunct treatment, bupropion, negative symptoms, schizophrenia

## Abstract

Antipsychotic treatment has been documented as the mainstay for the management of schizophrenia. Evidence in literature has suggested that the management of negative symptoms of schizophrenia continues to be a treatment challenge. Therefore, residual negative symptoms can become more pervasive and visible after the treatment of positive symptoms, leading to an impaired marked deficit in the vital daily functions of patients. We present a case series of three patients with a past psychiatric history of schizophrenia who presented to the psychiatric emergency with acute symptoms of schizophrenia. Following antipsychotic treatment, all these patients showed improvement of positive symptoms, however, profound negative symptoms of schizophrenia became visible. The negative symptoms include anhedonia, amotivation, alogia, affective flattening, and passive social withdrawal. We added bupropion to manage the negative symptoms, and all three patients achieved a good treatment response. This case series suggests that the anti-depressive effects of bupropion might be a valuable treatment option in the treatment of negative symptoms of schizophrenia.

## Introduction

Schizophrenia is a complex psychiatric disorder characterized by positive symptoms, such as delusions, hallucinations, disorganized speech, and disorganized behavior, and negative symptoms, such as anhedonia (lack of interest), amotivation, alogia, flat affect, and social withdrawal [1]. The negative symptoms are often residual and more difficult to treat because the positive symptoms are predominant and take precedence in the disease. About 20%-40% of patients with schizophrenia have persistent negative symptoms [2]. In the context of the chronic course of schizophrenia, persistent negative symptoms cause poor educational attainment, socio-occupational dysfunction, reduced ability of independent living, and poor quality of life [3].

The etiology of negative symptoms can be conceptualized as primary or secondary. Primary negative symptoms are disease-related and present during and between episodes of symptom exacerbation of schizophrenia. Secondary negative symptoms are non-disease-related, caused by medication side effects, other non-schizophrenia-related psychiatry problems, such as depression and social deprivation, or personality disorders and other medical disease-related factors [2]. Primary and secondary negative symptoms of schizophrenia may co-exist. They may also be non-persistent or appear for a shorter duration when compared to primary negative symptoms [2]. Hence, it is difficult to differentiate primary negative symptoms from secondary negative symptoms, which, in turn, poses a challenge for clinicians. Furthermore, primary negative symptoms do not generally respond well to currently available antipsychotic treatments, therefore, limited treatment options are available for negative symptoms of schizophrenia, making their management more challenging [1].

Second-generation antipsychotics are mostly effective in the treatment of positive symptoms of schizophrenia but the treatment options for persistent negative symptoms remain unknown, unclear, and therapeutically unmet [4-5]. Several case studies and trials document varying degrees of success in treating the negative symptoms of schizophrenia with several drugs, including bupropion, buspirone, memantine, methylphenidate, asenapine, amantadine, amisulpride, cariprazine, selegiline, and other antidepressants [4-13]. Among the atypical antipsychotic agents, cariprazine and amisulpride have been shown to decrease the negative symptoms [7,9]. Other drugs, like memantine, methylphenidate, and amantadine, and antidepressants like selegiline and bupropion had been shown to alleviate the negative symptoms when used in conjunction with antipsychotics as augmentation therapy [7,9,11,13]. The use of bupropion alone has shown mixed efficacy [4-5]. One study demonstrated no significant effect on the Scale for Assessment of Negative Symptoms (SANS) score on patients with negative symptoms [4] while another study showed five patients with negative depressive symptoms who significantly improved [12].

Evidence in literature has shown several drugs of various classes to have had a positive response in alleviating the negative symptoms of schizophrenia [14]. This provides evidence that the etiologies of negative symptoms vary, as it does from patient to patient. The development of negative symptoms of schizophrenia is multifactorial, which includes structural, neurobiological, environmental, and psychosocial symptoms [2]. The etiological factors identified and listed that may contribute to the development and evolution of negative symptoms are genetic contributions, prenatal events, and poor premorbid adjustment [15].

We present a case series with three patients, who on admission presented with acute symptoms of schizophrenia. Following antipsychotic treatment, all these patients showed a clinically significant improvement of positive symptoms, however, they exhibited profound negative symptoms of schizophrenia and were treated with bupropion. Treatment response was measured via the Positive and Negative Syndrome Scale (PANSS), which consists of 30 items and is the most widely used to measure the severity of illness in schizophrenia.

## Case presentation

Case 1

The patient was a 57-year-old man with a past psychiatric diagnosis of schizophrenia since the age of 19 years and a past medical history of pre-diabetes and asthma. The patient has had several past hospitalizations with an irregular outpatient follow-up and non-compliance to haloperidol decanoate 100 mg intramuscular (IM) monthly for the past three months. He has had past medication trials with olanzapine and haloperidol with haloperidol working best for him. He denied the use of any illicit substances for the past 20 years; however, he reported smoking half a pack of cigarettes a day with last use prior to this hospitalization. His reported psychosocial stressors were homelessness, unemployment, poor financial support, and poor social support.

He was brought in by emergency medical services (EMS) due to disorganized behavior, reportedly walking on the streets in his underwear. During the initial inpatient evaluation, he was psychotic and exhibited paranoid and persecutory delusions. He appeared internally preoccupied and reported auditory hallucinations. His thought process was disorganized, illogical with frequent derailments. He was disorganized in his behavior evidenced by walking around naked on the unit and laughing inappropriately. He exhibited a flat affect and was emotionally withdrawn and guarded. He was disheveled with poor grooming and hygiene. He reported his mood as “sad,” however, denied suicidal ideation, intent, or plan at the time of evaluation. He also denied homicidal ideation, intent, or plan at the time of evaluation. His PANSS assessment revealed a score of 102.

The patient’s treatment included haloperidol 5 mg PO twice daily (BID) followed by haloperidol decanoate 150 mg IM for psychosis and benztropine 1 mg PO BID for extrapyramidal symptom prevention. Haloperidol was chosen over a second-generation antipsychotic because the patient had responded well to haloperidol in the past. Over the next three weeks, the patient’s disorganized behavior improved. The thought process became more logical and organized. Delusional thoughts also improved, and he denied auditory hallucinations, however, he continued to exhibit negative symptoms, which became more prominent. He displayed a blunted affect and was socially and emotionally withdrawn. He also exhibited anhedonia and amotivation, was not engaging with staff, and did not attend groups or activities. He also reported “feeling sad.” Bupropion XL 150 mg PO daily was added to his treatment regimen. Over the next two weeks, his negative symptoms improved significantly. The patient was more visible in the unit, was noted to be interacting with staff and peers, and was able to express emotions. He also started attending groups/activities and was reaching out to family members. On week five, his PANSS score significantly improved to a score of 48 prior to discharge. He was discharged on haloperidol decanoate 150 mg IM monthly, bupropion XL 150 mg PO daily, and benztropine 1 mg PO daily.

Case 2

The patient was a 29-year-old man with a past psychiatric diagnosis of schizophrenia since the age of 20 years but no reported history for the use of substances or past medical history. The patient has had several past psychiatric hospitalizations with noncompliance to risperidone.

The patient was admitted on account of an aura sensation, “ear whooshing noise,” and paranoid delusions that people around him are laughing at him and creating these noises. He also exhibited persecutory delusions stating that people were constantly stalking him. He appeared internally preoccupied, with a disorganized thought process that was illogical and non-goal directed. He reported auditory hallucinations but was not able to elaborate. He was disorganized in his behavior, exhibited a flat affect, emotional withdrawal, and was guarded. He also exhibited intermittent crying spells. He was disheveled with poor grooming and hygiene. He reported his mood as “sad”; however, he denied suicidal ideation, intent, or plan at the time of evaluation. He also denied homicidal ideation, intent, or plan. His PANSS assessment revealed a score of 97 on admission.

The patient was treated with risperidone 2 mg PO BID for psychosis. He was also started on escitalopram 10 mg PO daily for depression. The patient agreed to take paliperidone palmitate. The initial dose of 234 mg IM and a booster dose of 156 mg IM was given five days apart. The patient reported extrapyramidal symptoms, which subsided with benztropine 1 mg PO BID. Over the next two weeks, the patient responded well to treatment for his auditory hallucinations and paranoid delusions; however, his depressive mood became more prominent. He was socially isolating self, less verbal, with flattening of affect. Escitalopram 10 mg PO daily was discontinued, and bupropion XL 150 mg PO daily was started for negative symptoms of schizophrenia. Within two weeks, his negative symptoms improved significantly. The patient became pleasant, more verbal, and engaging with staff and peers. He exhibited a brighter, appropriate, and reactive affect along with the ability to engage in meaningful conversations without frustration and crying spells. He was seen actively participating in groups and activities. He also endorsed hope about life and the future. At week four, his PANSS score improved to a score of 52 prior to his discharge. He was discharged on paliperidone palmitate 156 mg IM monthly, bupropion XL 150 mg PO daily, and benztropine 1 mg PO daily.

Case 3

The patient was a 49-year-old man with a past psychiatric history of schizophrenia since the age of 23 years and no pertinent past medical history. The patient was brought in by EMS activated by his brother on account of auditory hallucinations and medication non-compliance. As per the patient’s brother, the patient has had several hospitalizations in the past. The patient had tried risperidone in the past, which helped, however, currently he was on aripiprazole but not compliant. The brother stated that the patient gradually became more isolated, did not enjoy reading, watching basketball, or having conversations like he used to. When not on medications, the patient was talking to self and acting irrationally, walking and shouting at people on the streets, and very irritable. The brother denied any history of illicit substance use but reported that the patient smoked about half a pack a day.

During his initial evaluation in the inpatient unit, he appeared guarded and was a poor historian. He exhibited paranoid and persecutory delusions, stating that people are watching his house through cameras. He appeared very suspicious and was refusing to talk to staff on the unit. He reported auditory hallucinations and was observed having conversations with self. He endorsed irritability, reported poor sleep, but was unable to provide details. His thought process was disorganized, illogical, and non-goal directed. He appeared oddly groomed and disheveled. He reported his mood as “depressed” but denied suicidal ideation, intention, or plan at the time of evaluation. He also denied homicidal ideation, intent, or plan. The patient needed frequent redirections to engage with the writer. His PANSS assessment revealed a score of 99 on admission.

The patient was treated with risperidone 2 mg PO BID for psychosis because the patient has responded well to risperidone in the past. The patient was also started on sertraline 25 mg to target his depressive symptoms. In view of his non-compliance, the patient was offered a long-acting injectable, paliperidone palmitate, to which he agreed. He was given the first initiation dose of paliperidone palmitate 234 mg IM and a booster dose of 156 mg IM five days apart. The patient showed gradual improvement in his auditory hallucinations, persecutory delusions, and appeared less internally preoccupied; however, he remained unexpressive with blunted affect, isolative, secluded self to his room, and did not engage with staff or peers. He refused to go for group activities and was sleeping most of the day. The treatment team decided to start a trial of bupropion XL 150 mg PO daily for his negative symptoms, and sertraline was discontinued. The patient gradually started to show improvement of his negative symptoms as well. He was less depressed, less isolative, was more visible in the unit, and was observed attending group activities. The patient also requested a book to read. Over the course of three weeks following bupropion, more improvement was seen in his symptoms. At week five, his PANSS score significantly improved to a score of 39 prior to his discharge. He was discharged on paliperidone palmitate 156 mg IM monthly and bupropion XL 150 mg PO daily.

## Discussion

Schizophrenia is a chronic mental illness that enervates and devitalizes a person with disturbances in thoughts, perceptions, social attachments, and daily functioning [1]. In addition to delusions and hallucinations, individuals may have trouble organizing their speech and thoughts with behaviors that seem out of touch with reality. These are categorized as positive symptoms of schizophrenia and, when present, are referred to as acute symptoms of schizophrenia. The negative symptoms of schizophrenia are more subtle, more complex, and more difficult to differentiate because they can mimic various other psychiatric and medical illnesses. There are five classic negative symptoms identified and assessed in schizophrenia - alogia, affective blunting, avolition-apathy, anhedonia-asociality, and attentional impairment [16]. Negative symptoms are usually the most common first symptom of schizophrenia and can occur at any point in the course of its illness [1]. They may appear before experiencing the first acute psychotic episode, which is often referred to as the prodromal symptoms of schizophrenia, and they may also appear suppressed during an acute phase that predominates with positive symptoms of schizophrenia [1,17-18]. Negative symptoms can also be concomitant during an acute psychotic phase of schizophrenia and can remain chronic, residual, pervasive, and more debilitating during its illness [1]. The negative symptoms make people socially withdraw, isolate themselves to a house or room, less likely to initiate conversations, feel uncomfortable around people, lose self-appreciation about their appearance and personal hygiene, and lose interest and motivation in life, activities, and relationships including sex. These symptoms account for a large part of the long-term disability and poor functional outcomes in patients with schizophrenia [1]. In the above case series, our patients exhibited these classic negative symptoms leading to impaired social and personal outcomes, which without treatment would have been not only debilitating to the patient but also a debilitating burden to family and society.

Antipsychotic medications have been around for decades and have been the mainstay in the management of individuals diagnosed with schizophrenia; however, in terms of addressing the significant functional impairments that are associated with negative symptoms of schizophrenia, much needs to be done [4]. It is well-known that antipsychotic medications can successfully treat the positive symptoms of schizophrenia. Similarly, it is also known that most currently available antipsychotics have limited effects on the negative symptoms of schizophrenia, and to date, there is no agent approved by the FDA for the treatment of negative symptoms [15]. This unmet therapeutic need potentially impacts the daily functional outcomes of people with schizophrenia and could also reduce compliance, thereby imposing a greater risk of relapse. For this reason, various medications have been studied and evaluated as an adjunct to augment the effects of antipsychotic medications on negative symptoms. Given the considerable impact that negative symptoms can cause on its clinical and functional outcome and the challenges to differentiate primary from secondary negative symptoms that could closely mirror symptoms of depression, most clinical trials have strategized adding an adjunct antidepressant to the routine antipsychotic regimen [19]. Some clinical trials have demonstrated that adding bupropion to the routine antipsychotic regimen of patients with schizophrenia does not cause any significant improvement of negative symptoms [7], however, some other studies have demonstrated the opposite, where bupropion treatment was associated with improvement in negative symptoms with greater stability of psychotic and depressive symptoms [13]. A few clinical trials studied adding a second antipsychotic i.e. Abilify or Olanzapine [20-21], believing it may have an impact on the mood, particularly on depressive or negative symptoms because of its properties of mood stabilization, yet controversy continues to exist in terms of its effectiveness.

Preclinical and clinical data have shown that bupropion’s mechanism of action is via the dual inhibition of norepinephrine and dopamine reuptake (NDRI), constituting a novel and unique mechanism of antidepressant action with efficacy comparable to that of other antidepressants [22]. Bupropion has no effect on the serotonergic system. Hence, the most common observable side effects related to the serotonin reuptake inhibitor or serotonin-norepinephrine reuptake inhibitor antidepressants, such as weight gain, sexual dysfunction, or sedation, are not associated with bupropion use. This, therefore, can be a superior drug in comparison to the other anti-depressive agents for preventing avolition-apathy because it does not contribute to or mimic any secondary negative symptoms. Due to the uniqueness of its NDRI mechanism, bupropion has been assessed for use in several on- and off-label indications [23]. In addition to its use in the treatment of major depressive disorder, it has been used for the treatment of smoking cessation, attention deficit hyperactivity disorder, obesity, and bipolar depression [23]. Bupropion has been FDA-approved for major depressive disorder, seasonal affective disorder, and smoking cessation [24]. The atypical mechanism of actions of bupropion is believed to provide further benefit over selective serotonin reuptake inhibitors in its ability to improve anhedonia, fatigue, motivation, and focus retention [22]. Animal studies confirmed that acute administration of bupropion could decrease the firing of dopamine neurons in the brainstem in a dose-dependent manner, and this inhibitory effect may be associated with a unique clinical profile for improving negative symptoms in schizophrenia [25-26]. The mechanism by which Bupropion may stabilize negative symptoms among patients diagnosed with schizophrenia is unknown and needs more investigation on human models.

The goal of this case series was to show that patients in the three clinical presentations of negative symptom schizophrenia responded very well to bupropion. All three patients in our case series presented with acute symptoms of schizophrenia, were treated with antipsychotic medications, and showed a good therapeutic response to their positive symptoms, but they all experienced prominent negative symptoms. Adding bupropion to the regimen provided a clinically significant reduction in negative symptoms evidenced by an improvement in social and personal functioning as well as a significant reduction of the PANSS scores which indicated a reduction in the severity of illness. They were able to reach their individual baseline level of functioning, which likely will lead to an improvement in their quality of life. Given the clinical uncertainty and the challenge to differentiate between primary and secondary negative symptoms, the mechanism by which these individuals responded to Bupropion remains unclear. Because bupropion is FDA approved for major depressive disorder, it can be easily hypothesized that if Bupropion is targeting mood symptoms in schizophrenia and if the above patients were experiencing coexisting secondary negative symptoms of schizophrenia then the improvement in negative symptoms in this study could possibly be by improvement of mood rather than the core negative symptoms of schizophrenia. However, a possibility that the clinical improvement of adjunct bupropion was targeted on the primary core negative symptoms of schizophrenia rather than the mood could also exist. 

There are limited studies on bupropion as an adjunct for the treatment of negative symptoms of schizophrenia and more studies with reproducibility are needed to affirm its effectiveness. This study was noted to have its limitations, some of which are limited sample size, unavailability of more statistically relevant data, and limited use of clinical assessments where only the PANSS score was obtained, not the Marder PANSS scores. Also, clinical assessments, i.e., SANS, Brief Negative Symptom Scale (BNSS), and Negative Symptom Assessment NSA-16, were not used in this study, and an inability to follow up to assess compliance and functional outcomes following discharge are additional limitation factors.

## Conclusions

Our case series showed a good clinical response with adjunct bupropion in the management of the negative symptoms of schizophrenia. For now, no single co-treatment strategy has been sufficiently proven effective or recommended for the treatment of patients with negative symptoms of schizophrenia. Evidence in literature has shown that adjunctive efficacy is limited by methodological issues suggesting that higher-quality trials and patient-based meta-analyses are needed.
